# Supplementary material for: Direct-acting antiviral resistance of Hepatitis C virus is promoted by epistasis
Source: Nat Commun. 2023 Nov 17;14:7457. doi: 10.1038/s41467-023-42550-6 (PMC10656532; doi:10.1038/s41467-023-42550-6)
Supplement: Supplementary file 1 — Supplementary Information [file 41467_2023_42550_MOESM1_ESM.pdf]

Supplementary Information

Direct-acting antiviral resistance of Hepatitis C virus is promoted by epistasis

Table of contents

Supplementary Figures 1-8

Supplementary Tables 1-7

Supplementary References

Supplementary Figures

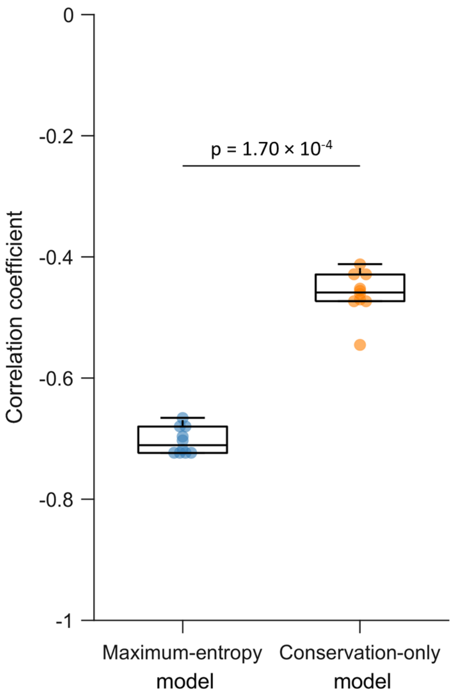

Supplementary Figure 1: **Robustness of the correlation observed between model energies and experimental fitness values. Results are shown for the maximum-entropy model that considers epistatic interaction and for the conservation-only model that ignores epistasis.** The sequence data used for inferring each model was generated by a standard bootstrap procedure using ten samples. The reported p-value was calculated using the two-sided Mann-Whitney test ($n_1 = 9$ SC-DRMs and $n_2 = 11$ remaining DRMs). Source data are provided as a Source Data file.

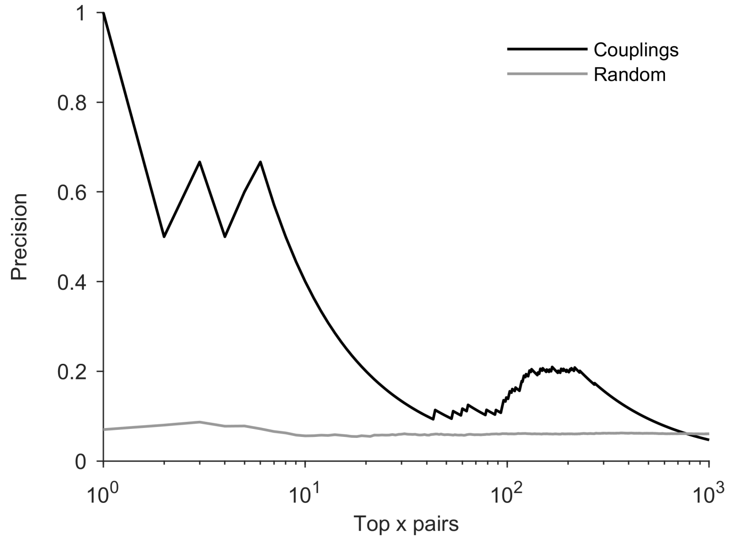

Supplementary Figure 2: **Top ranked pairs of mutations based on the strength of their couplings (ranked by the values of -J from Eq. 1) are more likely to involve DRMs as compared to pairs picked randomly.** Precision is the proportion of top x pairs that involve at least one DRM based on the model couplings (shown in black) or picking pairs randomly (shown in gray; results averaged over 10 random realizations). Source data are provided as a Source Data file.

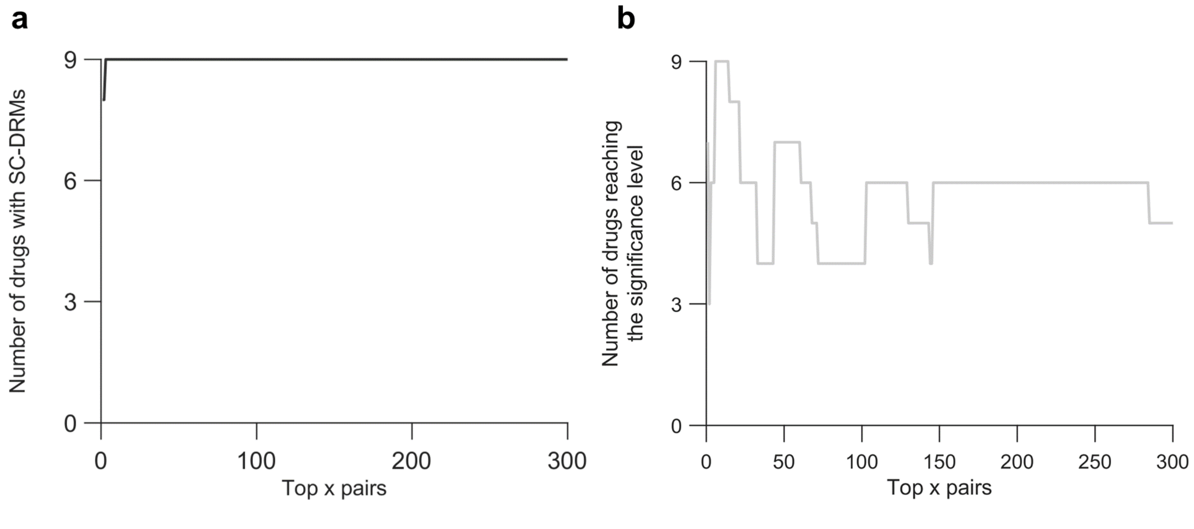

Supplementary Figure 3: **Robustness of the enrichment of SC-DRMs in each drug (Figure 4) to the number of top-coupled pairs of mutations used to define SC-DRMs.** (**a**) Number of drugs with at least one SC-DRM vs. top x (x $\in$ [0, 300]) pairs of mutations used to define SC-DRMs. Pairs of mutations were ranked based on the couplings of the inferred model (ranked by the values of -J from Eq. 1), and DRMs appearing among the top x pairs of mutations were considered to be SC-DRMs. (**b**) Number of drugs for which the p-value associated with the number of SC-DRMs reached the significance level (p-value < 0.05) vs. top x (x $\in$ [0, 300]) pairs of mutations used to define SC-DRMs. Statistically significant enrichment of SC-DRMs was observed for the majority of drugs ($\geq$5) for almost all values of x. Here, the p-value measures the probability of observing by a random chance at least the observed number of SC-DRMs among all DRMs associated with a drug (see Methods for details). DRMs against each drug are listed in Table 1.[1–4] Source data are provided as a Source Data file.

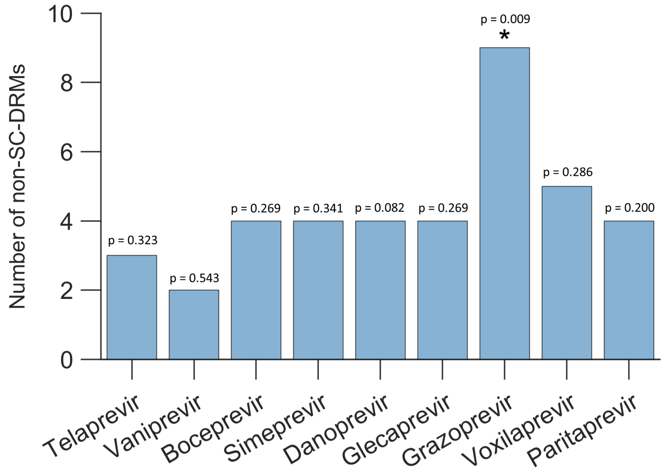

Supplementary Figure 4: **Statistical significance of the number of non-SC-DRMs associated with each drug.** Non-SC-DRMs are DRMs not appearing in the top 300 pairs of mutations based on the couplings of the inferred model (ranked by the values of -J from Eq. 1). The p-value measures the probability of observing by a random chance at least the observed number of non-SC-DRMs among all DRMs associated with a drug (see Methods for details). Statistical significant results (p-value < 0.05) are marked with a star on the top of each bar. Source data are provided as a Source Data file.

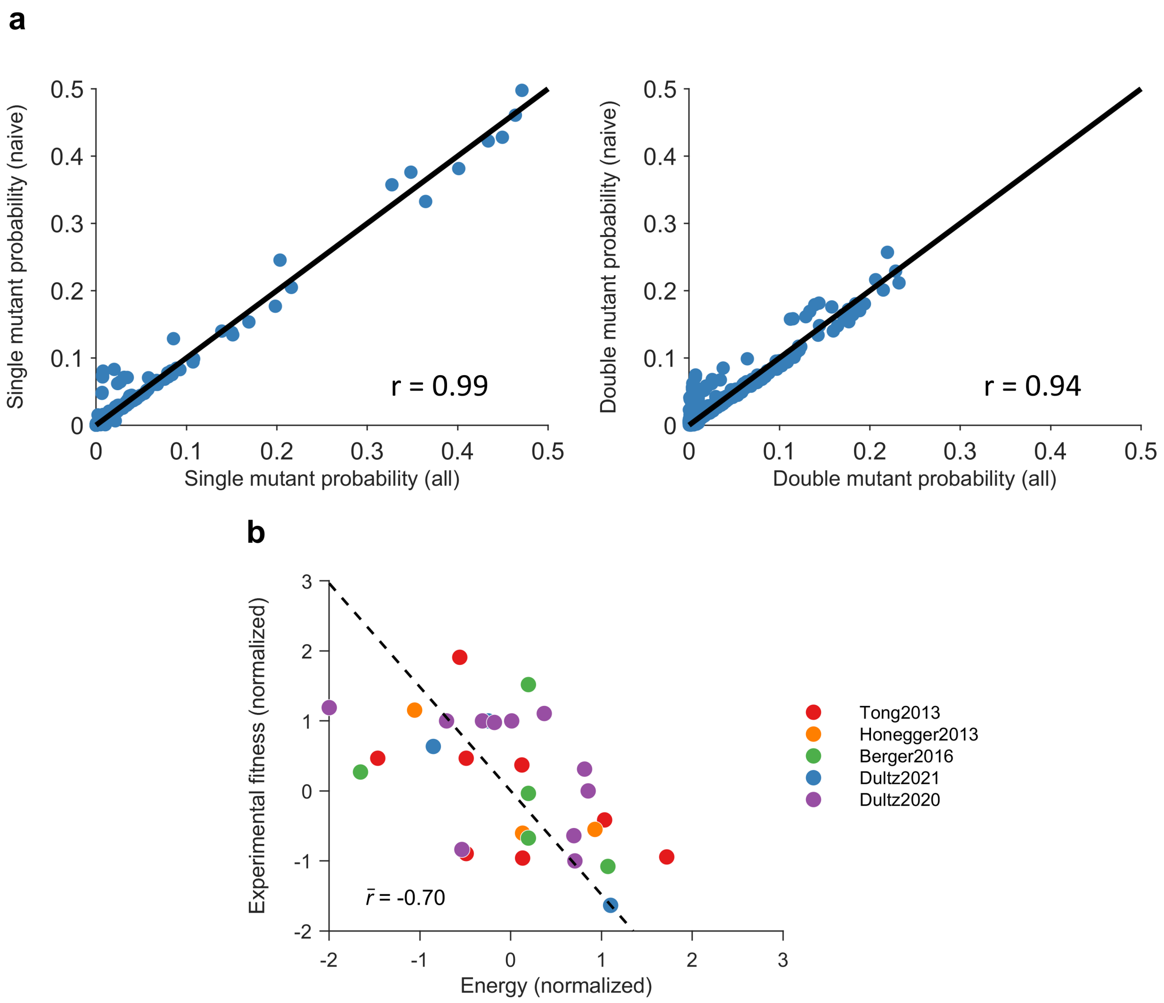

Supplementary Figure 5: **Comparison of statistical properties and model predictions based on complete data with those based on a subset of drug-naïve patients.** (**a**) Correlation of single mutant probabilities (left panel) and double mutant probabilities (right panel) between sequences from all patients (7370 sequences) and the subset of drug-naïve patients (5877 sequences). (**b**) Correlation between model predicted energies and experimental fitness measurements compiled from different studies (mentioned in the legend). Normalization of both fitness measurements and predicted model energies was performed by subtracting the mean from each data set and dividing by its standard deviation. Source data are provided as a Source Data file.

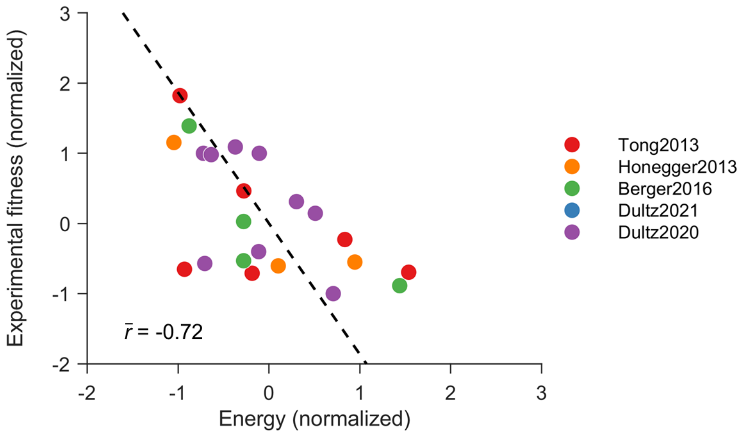

Supplementary Figure 6: **Correlation between the model predicted energies and 36 experimental fitness measurements that are associated with DRMs.** These fitness measurements were compiled from different studies that are mentioned in the legend. Normalization of both fitness measurements and predicted model energies was performed by subtracting the mean from each data set and dividing by its standard deviation. Source data are provided as a Source Data file.

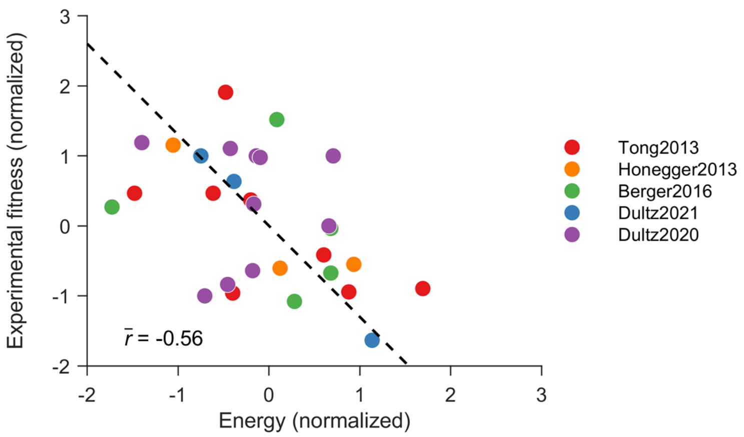

Supplementary Figure 7: **Correlation between the sequence energy obtained from newly inferred model and in-vitro infectivity measurements.** This model was inferred by including all sequences (9683 sequences) and weighted each sequence without patient information (2167 sequences) as 1. These fitness measurements were compiled from different studies that are mentioned in the legend. Normalization of both fitness measurements and predicted model energies was performed by subtracting the mean from each data set and dividing by its standard deviation. Source data are provided as a Source Data file.

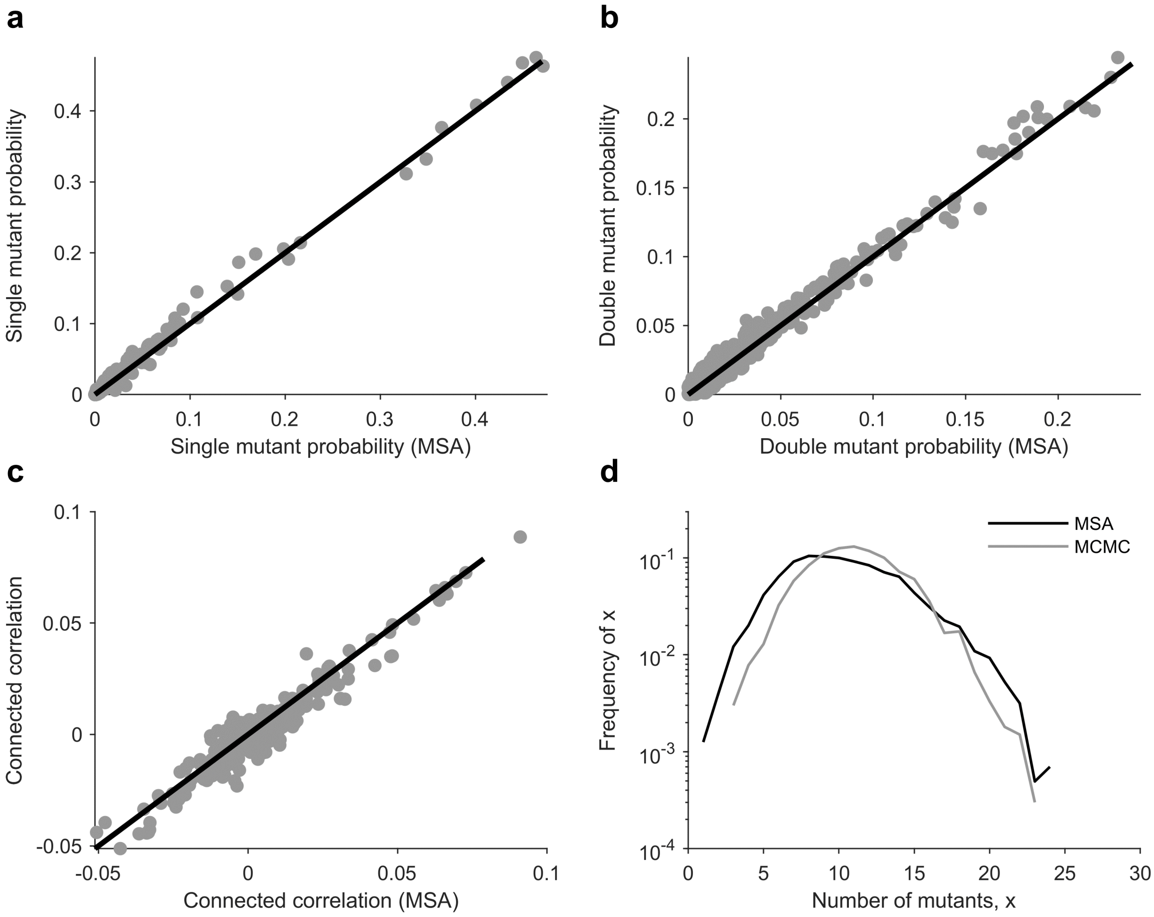

Supplementary Figure 8: **Statistical validation of the inferred model for the HCV NS3 protein.** Comparison of the (**a**) single mutant probabilities, (**b**) double mutant probabilities, (**c**) connected correlations, and (**d**) distribution of the number of mutants per sequence obtained from the MSA and those predicted by the inferred model. Connected correlations represent correlations which cannot be explained by lower order mutant probabilities and is given by $f_{ij}(a,b) - f_i(a)f_j(b)$, where $f_i(a)$ is the probability of observing mutant a at residue i while f_{ij} is the probability of simultaneously observing mutants a and b at residues i and j respectively. The number of mutants per sequence is the number of amino acids that are different in a sequence from those of the consensus sequence (sequence constructed with the most-frequent amino acid at each residue). Samples were generated from the inferred model using the Markov Chain Monte Carlo method.[5] Source data are provided as a Source Data file.

Supplementary Tables

Supplementary Table 1: **List of NS3 residues in each co-evolutionary sector inferred using RocaSec[6,7]**

Sectors	Residues in sector
1	29, 40, 51, 53, 54, 55, 64, 66, 67, 80, 86, 87, 94, 121, 122, 124, 150, 151, 174, 178, 185, 186, 187, 192, 197, 200, 247, 248, 264, 295, 315, 318, 329, 333, 334, 337, 338, 358, 370, 371, 372, 379, 383, 394, 395, 417, 418, 484, 485, 490, 517, 518, 586, 593, 594, 610, 614, 615
2	36, 40, 42, 51, 52, 53, 54, 55, 64, 69, 70, 71, 80, 87, 94, 124, 151, 155, 156, 157, 170, 171, 174, 185, 186, 187, 192, 197, 224, 225, 247, 248, 264, 295, 315, 318, 337, 338, 340, 341, 358, 370, 371, 372, 379, 383, 384, 386, 394, 395, 401, 402, 417, 418, 484, 485, 517, 518, 557, 558, 593, 594, 609, 610, 614, 615
3	452, 453, 459, 460, 461, 462, 463, 468, 471, 511, 512
4	29, 36, 40, 42, 51, 52, 53, 54, 55, 64, 66, 80, 86, 87, 91, 124, 150, 151, 155, 156, 157, 170, 171, 178, 192, 197, 200, 224, 225, 247, 248, 295, 318, 329, 333, 334, 337, 338, 340, 341, 370, 371, 372, 379, 383, 384, 386, 394, 395, 401, 402, 417, 418, 484, 485, 490, 505, 517, 518, 541, 576, 586, 593, 594, 609, 610
5	1, 2, 3, 4, 5, 6, 11, 12, 15, 19, 24, 29, 36, 42, 51, 52, 53, 54, 55, 64, 67, 80, 87, 91, 124, 150, 151, 155, 156, 157, 170, 171, 192, 196, 200, 213, 329, 337, 338, 340, 341, 357, 370, 371, 401, 402, 403, 404, 469, 470, 472, 484, 485, 517, 518, 535, 570, 614, 615
6	2, 3, 4, 5, 6, 11, 12, 15, 19, 24, 29, 36, 42, 51, 52, 53, 54, 55, 64, 67, 80, 86, 87, 91, 121, 122, 124, 150, 151, 155, 156, 157, 170, 171, 192, 196, 197, 200, 318, 329, 333, 334, 337, 338, 340, 341, 357, 370, 371, 383, 386, 401, 402, 403, 404, 417, 418, 469, 470, 472, 484, 485, 490, 517, 518, 586, 614, 615
7	29, 40, 51, 53, 54, 55, 66, 67, 86, 87, 91, 94, 121, 122, 124, 150, 151, 178, 185, 186, 187, 192, 196, 197, 200, 224, 225, 247, 248, 252, 295, 309, 310, 315, 318, 333, 334, 337, 338, 357, 358, 370, 371, 379, 384, 394, 395, 399, 401, 402, 403, 404, 469, 470, 472, 484, 485, 517, 518, 557, 558, 576, 586, 593, 594

Supplementary Table 2: **List of NS3 residues in experimentally-known biochemical domains**

**Biochemical Domains**	**Acronyms**	**Residues involved in each domain**	**References**
NS4A interface for protease activation	NS3-NS4A-Pro-Act	1, 2, 3, 4, 5, 6, 7, 8, 9, 10, 11, 12, 13, 14, 15, 16, 17, 18, 19, 20, 21, 22	8
NS3-NS4A membrane association	NS3-NS4A-Mem-Asso	10, 11, 12, 13, 14, 15, 16, 17, 18, 19, 20, 21, 22, 23, 24	9
NS5A hyper-phosphorylation	NS5A-Hyper-Phos	1, 2, 3, 4, 5, 6, 7, 8, 9, 10, 139	10, 11
Motif important for enzymatic and helicase activities in NS3	NS3-Motif-Enz-Heli	460, 461, 462, 463, 464, 465, 466, 467	12
Intra-dimer interface in NS3 helicase	NS3-Intra-Dimer-Int	435, 436, 437, 438, 439, 440, 441, 442, 443, 444, 445, 446, 447, 448, 449, 450, 451, 452, 453, 477, 478, 479, 480, 481, 482, 483, 484, 485, 486, 487, 488, 524, 525, 526, 527, 528, 529, 530, 531, 532, 533, 534, 535, 536, 545, 546, 547, 548, 549, 550, 551, 552, 553, 584, 585, 586, 587, 588, 589, 590, 591	13

Supplementary Table 3: **List of top-coupled mutations that are predicted to be compensatory for SC-DRMs**

**SC-DRM**	**Total number of selected sequence backgrounds**	**Associated compensatory mutation**	**Percentage of sequence backgrounds where mutation has a compensatory effect**
41R	7362	168E	100%
168E	7318	41R	100%
170V	7011	174S	73.2%
80K	4553	615V	61.1%
122G	6953	174S	60.2%
122C	7207	174S	51.8%
55A	7250	40T	29.6%
80K	4553	91S	22.7%
36L	7292	197Y	11.5%
122C	7207	197Y	10.3%
122C	6953	318T	10%

Each row shows the SC-DRM, the number of MSA sequences lacking the SC-DRM, the associated compensatory mutation (among top 300 pairs of mutations with large values of $-J_{ij}$), and the percentage of sequences where the associated mutation was found to compensate for the SC-DRM.

Supplementary Table 4: **List of DRM-associated residues in the binding residues of drugs with known structure**

Drug	Residues with DRMs in binding residues
Danoprevir	**41**, 43, **55**, **80**, 132, 138, 155, 156, 158, **168**
Vaniprevir	**41**, 43, **55**, **80**, 132, 138, 155, 156, 158, **168**
Telaprevir	**41**, 43, **55**, 132, 138, 155, 156, 158, **168**
Grazoprevir	**41**, 43, **55**, 56, 132, 138, 155, 156, 158, **168**

SC-DRMs are shown in **bold** and DRMs from other drugs exclusively are underlined.

Supplementary Table 5: **List of binding residues of drugs with known structures.**

Drug	Binding residues
Danoprevir	41, **42**, 43, 55, **57**, **58**, **78**, **79**, 80, **81**, **123**, 132, **135**, **136**, **137**, 138, **139**, **154**, 155, 156, **157**, 158, **159**, 168
Vaniprevir	41, **42**, 43, 55, **57**, **58**, **78**, **79**, 80, **81**, **123**, 132, **135**, **136**, **137**, 138, **139**, **154**, 155, 156, **157**, 158, **159**, 168
Telaprevir	41, **42**, 43, 55, **57**, **81**, **123**, 132, **135**, **136**, **137**, 138, **139**, **154**, 155, 156, **157**, 158, **159**, 168
Grazoprevir	41, **42**, 43, 55, 56, **57**, **58**, **78**, **81**, **123**, 132, **135**, **136**, **137**, 138, **139**, **154**, 155, 156, **157**, 158, **159**, 168

Residues that are not associated with any DRMs are shown in **bold.** Of these, residues that are associated with strong compensatory interactions based on our model (top 300 pairs of mutations with large values of $-J_{ij}$) are also underlined.

Supplementary Table 6: **Efficacy of each NS3-targeting drug and the number of SC-DRMs associated with them**

Drug	DAA regimen	Number of SC-DRMs	Weighted average of SVR rates (%)	Total number of patients	References
Telaprevir	Telaprevir	3	68.5	992	14–16
Vaniprevir	Vaniprevir	2	73.6	110	17,18
Boceprevir	Boceprevir	5	63.7	947	19,20
Simeprevir	Simeprevir	6	71.8	1132	21–24
	Simeprevir-sofosbuvir		91.8	425	25,26
Danoprevir	Danoprevir	2	72.0	1264	27–29
	Danoprevir-sofosbuvir		100	58	30
Glecaprevir	Glecaprevir-pibrentasvir	5	99.4	703	31
Grazoprevir	Elbasvir-grazoprevir	7	96.6	1297	32
Voxilaprevir	Sofosbuvir-velpatasvir-voxilaprevir	8	95.2	461	33,34
Paritaprevir	Ombitasvir-paritaprevir-ritonavir / Ombitasvir-paritaprevir-ritonavir-dasabuvir	4	96.8	5046	35

Supplementary Table 7: **Details of infectivity measurements obtained from each study (listed in Supplementary Data 1).**

Reference	Figure/Table	Type of experiment	Number of measurements
36	Table 1 and Figure 2	replicative capacity	22
37	Figure 6	replicative capacity	5
38	Table 4	replicative capacity	8
39	Figure 3f	replicative capacity	3
40	Supplementary Table H	replicative capacity	5

Supplementary References

1. Singer, J. B., Thomson, E. C., McLauchlan, J., Hughes, J. & Gifford, R. J. GLUE: A flexible software system for virus sequence data. *BMC Bioinformatics* **19**, 532 (2018).
2. Singer, J. *et al.* Interpreting viral deep sequencing data with GLUE. *Viruses* **11**, 323 (2019).
3. Sorbo, M. C. *et al.* Hepatitis C virus drug resistance associated substitutions and their clinical relevance: Update 2018. *Drug Resistance Updates* **37**, 17–39 (2018).
4. Romano, K. P., Ali, A., Royer, W. E. & Schiffer, C. A. Drug resistance against HCV NS3/4A inhibitors is defined by the balance of substrate recognition versus inhibitor binding. *Proceedings of the National Academy of Sciences* **107**, 20986–20991 (2010).
5. Ferguson, A. L. *et al.* Translating HIV sequences into quantitative fitness landscapes predicts viral vulnerabilities for rational immunogen design. *Immunity* **38**, 606–617 (2013).
6. Quadeer, A. A., Morales-Jimenez, D. & McKay, M. R. Co-evolution networks of HIV/HCV are modular with direct association to structure and function. *PLoS Computational Biology* **14**, 1–29 (2018).
7. Quadeer, A. A., Morales-Jimenez, D. & McKay, M. R. ROCASEC: A standalone GUI-based package for robust co-evolutionary analysis of proteins. *Bioinformatics* **36**, 2262–2263 (2019).
8. Tan, S.-L. & Lin, C. *Chapter 6*, 164–206 (Horizon Bioscience, 2006).
9. Brass, V. *et al.* Structural determinants for membrane association and dynamic organization of the hepatitis C virus NS3-4A complex. *Proceedings of the National Academy of Sciences* **105**, 14545–14550 (2008).
10. Koch, J. O. & Bartenschlager, R. Modulation of hepatitis C virus NS5A hyperphosphorylation by non-structural proteins NS3, NS4A, and NS4B. *Journal of Virology* **73**, 7138–7146 (1999).
11. Neddermann, P., Clementi, A. & De Francesco, R. Hyperphosphorylation of the hepatitis C virus NS5A protein requires an active NS3 protease, NS4A, NS4B, and NS5A encoded on the same polyprotein. *Journal of Virology* **73**, 9984–9991 (1999).
12. Kim, D. W., Kim, J., Gwack, Y., Han, J. H. & Choe, J. Mutational analysis of the hepatitis C virus RNA helicase. *Journal of Virology* **71**, 9400–9409 (1997).
13. Mackintosh, S. G. *et al.* Structural and biological identification of residues on the surface of NS3 helicase required for optimal replication of the hepatitis C virus. *Journal of Biological Chemistry* **281**, 3528–3535 (2006).
14. McHutchison, J. G. *et al.* Telaprevir with peginterferon and ribavirin for chronic HCV genotype 1 infection. *New England Journal of Medicine* **360**, 1827–1838 (2009).

15. Hézode, C. *et al.* Telaprevir and peginterferon with or without ribavirin for chronic HCV infection. *New England Journal of Medicine* **360**, 1839–1850 (2009).

16. Jacobson, I. M. *et al.* Telaprevir for previously untreated chronic hepatitis C virus infection. *New England Journal of Medicine* **364**, 2405–2416 (2011).

17. Manns, M. P. *et al.* Vaniprevir with pegylated interferon alpha-2a and ribavirin in treatment-naïve patients with chronic hepatitis C: A randomized phase ii study. *Hepatology* **56**, 884–893 (2012).

18. LLC, M. S. . D. A study of vaniprevir (MK-7009) in participants with chronic hepatitis C infection after participation in other vaniprevir studies (MK-7009-028). URL https://clinicaltrials.gov/ct2/show/NCT00943761.

19. Kwo, P. Y. *et al.* Efficacy of boceprevir, an NS3 protease inhibitor, in combination with peginterferon alfa-2b and ribavirin in treatment-naive patients with genotype 1 hepatitis C infection (SPRINT-1): an open-label, randomised, multicentre phase 2 trial. *The Lancet* **376**, 705–716 (2010).

20. Poordad, F. *et al.* Boceprevir for untreated chronic HCV genotype 1 infection. *New England Journal of Medicine* **364**, 1195–1206 (2011).

21. Jacobson, I. M. *et al.* Simeprevir with pegylated interferon alfa 2a plus ribavirin in treatment-naive patients with chronic hepatitis C virus genotype 1 infection (QUEST-1): a phase 3, randomised, double-blind, placebo-controlled trial. *The Lancet* **384**, 403–413 (2014).

22. Fried, M. W. *et al.* Once-daily Simeprevir (TMC435) with pegylated interferon and ribavirin in treatment-naïve genotype 1 hepatitis C: The randomized pillar study. *Hepatology* **58**, 1918–1929 (2013).

23. Asselah, T. *et al.* An open-label trial of 12-week simeprevir plus peginterferon/ribavirin (PR) in treatment-naïve patients with hepatitis C virus (HCV) genotype 1 (GT1). *PLoS ONE* **11**, 1–15 (2016).

24. Zeuzem, S. *et al.* Simeprevir increases rate of sustained virologic response among treatment-experienced patients with HCV genotype-1 infection: A phase IIb trial. *Gastroenterology* **146**, 430–441.e6 (2014).

25. Lawitz, E. *et al.* Simeprevir plus sofosbuvir, with or without ribavirin, to treat chronic infection with hepatitis C virus genotype 1 in non-responders to pegylated interferon and ribavirin and treatment-naive patients: the COSMOS randomised study. *The Lancet* **384**, 1756–1765 (2014).

26. Kwo, P. *et al.* Simeprevir plus sofosbuvir (12 and 8 weeks) in hepatitis C virus genotype 1-infected patients without cirrhosis: OPTIMIST-1, a phase 3, randomized study. *Hepatology* **64**, 370–380 (2016).

27. Marcellin, P. *et al.* Randomized controlled trial of danoprevir plus peginterferon alfa-2a and ribavirin in treatment-naïve patients with hepatitis C virus genotype 1 infection. *Gastroenterology* **145**, 790–800.e3 (2013).

28. Everson, G. *et al.* DAUPHINE: a randomized phase II study of danoprevir/ritonavir plus peginterferon alpha-2a/ribavirin in HCV genotypes 1 or 4. *Liver International* **35**, 108–119 (2015).

29. Feld, J. J. *et al.* Randomized study of danoprevir/ritonavir-based therapy for HCV genotype 1 patients with prior partial or null responses to peginterferon/ribavirin. *Journal of Hepatology* **62**, 294–302 (2015).

30. Pan, S. *et al.* Efficacy and safety of danoprevir plus sofosbuvir in GT 1, 2, 3, or 6 chronic hepatitis C patients with or without cirrhosis in China. *Medicine* **100** (2021).

31. Zeuzem, S. *et al.* Glecaprevir-pibrentasvir for 8 or 12 weeks in HCV genotype 1 or 3 infection. *New England Journal of Medicine* **378**, 354–369 (2018).

32. Ahmed, H. *et al.* Meta-analysis of grazoprevir plus elbasvir for treatment of hepatitis C virus genotype 1 infection. *Annals of Hepatology* **17**, 18–32 (2018).

33. Jacobson, I. M. *et al.* Efficacy of 8 weeks of sofosbuvir, velpatasvir, and voxilaprevir in patients with chronic HCV infection: 2 phase 3 randomized trials. *Gastroenterology* **153**, 113–122 (2017).

34. Bourlière, M. *et al.* Sofosbuvir, velpatasvir, and voxilaprevir for previously treated hcv infection. *New England Journal of Medicine* **376**, 2134–2146 (2017).

35. Wedemeyer, H. *et al.* Real-world effectiveness of ombitasvir/paritaprevir/ritonavir±dasabuvir±ribavirin in patients with hepatitis C virus genotype 1 or 4 infection: A meta-analysis. *Journal of Viral Hepatitis* **24**, 936–943 (2017).

36. Dultz, G. *et al.* Extended interaction networks with HCV protease NS3-4A substrates explain the lack of adaptive capability against protease inhibitors. *Journal of Biological Chemistry* **295**, 13862–13874 (2020).

37. Dultz, G. *et al.* Epistatic interactions promote persistence of NS3-Q80K in HCV infection by compensating for protein folding instability. *Journal of Biological Chemistry* **297** (2021).

38. Tong, X., Li, L., Haines, K. & Najera, I. Identification of the NS5B S282T resistant variant and two novel amino acid substitutions that affect replication capacity in hepatitis C virus-infected patients treated with mericitabine and danoprevir. *Antimicrobial agents and chemotherapy* **58**, 3105–3114 (2014).

39. Honegger, J. R. *et al.* Loss of immune escape mutations during persistent HCV infection in pregnancy enhances replication of vertically transmitted viruses. *Nature Medicine* **19**, 1529–1533 (2013).

40. Berger, K. L. *et al.* Resistance analyses of HCV NS3/4A protease and NS5B polymerase from clinical studies of deleobuvir and faldaprevir. *PLoS ONE* **11**, 1–16 (2016).
