# Supplementary material for: Direct-acting antiviral resistance of Hepatitis C virus is promoted by epistasis
Source: Nat Commun. 2023 Nov 17;14:7457. doi: 10.1038/s41467-023-42550-6 (PMC10656532; doi:10.1038/s41467-023-42550-6)
Supplement: Supplementary file 3 — Reporting Summary [file 41467_2023_42550_MOESM3_ESM.pdf]

nature portfolio

Corresponding author(s): MATTHEW MCKAY AND AHMED ABDUL QUADEER

Last updated by author(s): Oct 2, 2023

Reporting Summary

Nature Portfolio wishes to improve the reproducibility of the work that we publish. This form provides structure for consistency and transparency in reporting. For further information on Nature Portfolio policies, see our Editorial Policies and the Editorial Policy Checklist.

Statistics

For all statistical analyses, confirm that the following items are present in the figure legend, table legend, main text, or Methods section.

n/a	Confirmed	
☐	☒	The exact sample size (*n*) for each experimental group/condition, given as a discrete number and unit of measurement
☐	☒	A statement on whether measurements were taken from distinct samples or whether the same sample was measured repeatedly
☐	☒	The statistical test(s) used AND whether they are one- or two-sided<br>*Only common tests should be described solely by name; describe more complex techniques in the Methods section.*
☒	☐	A description of all covariates tested
☐	☒	A description of any assumptions or corrections, such as tests of normality and adjustment for multiple comparisons
☐	☒	A full description of the statistical parameters including central tendency (e.g. means) or other basic estimates (e.g. regression coefficient) AND variation (e.g. standard deviation) or associated estimates of uncertainty (e.g. confidence intervals)
☐	☒	For null hypothesis testing, the test statistic (e.g. *F*, *t*, *r*) with confidence intervals, effect sizes, degrees of freedom and *P* value noted<br>*Give P values as exact values whenever suitable.*
☐	☒	For Bayesian analysis, information on the choice of priors and Markov chain Monte Carlo settings
☐	☒	For hierarchical and complex designs, identification of the appropriate level for tests and full reporting of outcomes
☐	☒	Estimates of effect sizes (e.g. Cohen's *d*, Pearson's *r*), indicating how they were calculated

Our web collection on statistics for biologists contains articles on many of the points above.

Software and code

Policy information about availability of computer code

Data collection	No software was used to collect the data used in this study.
Data analysis	For inferring the NS3 1a maximum entropy fitness landscape model's parameters, the GUI-based software implementation of the MPF-BML method (https://github.com/ahmedaq/MPF-BML-GUI) was used.<br>For the co-evolutionary analysis of NS3 1a, the GUI-implementation of the robust co-evolutionary analysis approach, RocaSec (https://github.com/ahmedaq/RocaSec), was used.<br>For computing the distance between atoms in each protein structure and for drawing the structural figures, the PyMOL software (https://www.pymol.org) was used.<br>All statistical analyses in this work were performed using MATLAB R2021a. Data and scripts for reproducing the results are available at https://github.com/hangzhangust/HCV NS3.

For manuscripts utilizing custom algorithms or software that are central to the research but not yet described in published literature, software must be made available to editors and reviewers. We strongly encourage code deposition in a community repository (e.g. GitHub). See the Nature Portfolio guidelines for submitting code & software for further information.

Data

Policy information about availability of data

All manuscripts must include a data availability statement. This statement should provide the following information, where applicable:

- Accession codes, unique identifiers, or web links for publicly available datasets
- A description of any restrictions on data availability
- For clinical datasets or third party data, please ensure that the statement adheres to our policy

For inferring the maximum entropy based fitness landscape model, NS3 subtype 1a aligned sequences (coverage >= 99%) were downloaded from the publicly available HCV GLUE sequence database, http://hcv.glue.cvr.ac.uk.
For validation of the inferred NS3 1a fitness landscape model, the ex-vivo experimental fitness (infectivity) measurements were compiled from five literature reports.
Information of drug resistant mutations of nine NS3-specific drugs used for treating HCV genotype 1a infections were obtained from the HCV GLUE database (http://hcv.glue.cvr.ac.uk), as well as from two relevant literature studies.
All NS3 1a protein crystal structures (PDB ID: 4B6E, 3M5L, 3SU3, 3SV6, 3SUD) used in the analysis were obtained from the Protein Databank (https://www.rcsb.org).
For the drug efficacy analysis, efficacy data of nine NS3-specific drugs used for treating patients infected with HCV genotype 1a was compiled from 22 literature studies.
All data used in this work has been provided in the supplementary data files and is publicly available as of the date of publication. The infectivity measurements for NS3, used for correlating with predictions from the fitness landscape model, are included in Supplementary Data 1. Accession numbers of NS3 sequences used for inferring the model are listed in Supplementary Data 2. The mean escape time predicted by the in-host evolutionary model for each residue with DRMs is provided in Supplementary Data 3. Source data for all figures are provided with this paper. Any additional information related to the data reported in this paper is available from the lead contact upon request.

Research involving human participants, their data, or biological material

Policy information about studies with human participants or human data. See also policy information about sex, gender (identity/presentation), and sexual orientation and race, ethnicity and racism.

Reporting on sex and gender	This study does not involved any sex or gender information as this information does not influence the conclusions drawn by the analysis conducted in the study.
Reporting on race, ethnicity, or other socially relevant groupings	N/A
Population characteristics	All HCV NS3 sequences anlalyzed in this study were collected from patients infected with HCV genotype 1a.
Recruitment	N/A
Ethics oversight	N/A

Note that full information on the approval of the study protocol must also be provided in the manuscript.

Field-specific reporting

Please select the one below that is the best fit for your research. If you are not sure, read the appropriate sections before making your selection.

☐ Life sciences ☐ Behavioural & social sciences ☒ Ecological, evolutionary & environmental sciences

For a reference copy of the document with all sections, see nature.com/documents/nr-reporting-summary-flat.pdf

Ecological, evolutionary & environmental sciences study design

All studies must disclose on these points even when the disclosure is negative.

Study description	We employed a maximum-entropy-based method to infer an in silico model for the fitness landscape of the HCV NS3 protein using available sequences for genotype 1a. We then integrated the fitness landscape into a stochastic population genetics model of in-host viral evolution, which we employed to quantify the average time to escape from selective pressure of drugs targeting specific residues in NS3.
Research sample	All 9683 publicly available NS3 genotype 1a sequences with coverage >= 99% (access date: 2021/09/02) were downloaded from the HCV-GLUE database (http://hcv.glue.cvr.ac.uk). This data is meant to represent sequences of patients infected with HCV NS3 genotype 1a.
Sampling strategy	Only sequences with coverage >= 99% were downloaded from the database to control the data quality, i.e., to ensure minimum number of gaps in the downloaded sequences. No further criteria was used for selecting the samples.<br><br>In our study, n = 9683 represents all sequences available from patients infected with HCV genotype 1a. No sample size calculation

	was performed.<br><br>The one- and two-point statistics of the sequence data, which we use to infer computational models, has been shown to be robust to the change of number of sequences included for our analysis. So this sample size is sufficient to conduct our analysis.
Data collection	The data was downloaded directly from the HCV-GLUE database (http://hcv.glue.cvr.ac.uk) by the authors.
Timing and spatial scale	The data was collected from the database once at the date Sep-02-2021 that includes all the up-to-date HCV NS3 genotype 1a sequence data. This downloaded data includes sequences from multiple patients collected from 1997 - 2017, where the month of the samples, frequency and periodicity of sampling of this data is not provided.
Data exclusions	We conducted principal component analysis (PCA) of the pair-wise similarity matrix constructed from the sequence data to remove outlying sequences. We also excluded sequences that were not associated with any patients. We also excluded from this data fully-conserved residues, i.e., residues where no mutation was observed in any sequence. Thus, we excluded 2167 sequences in total to control the quality of the data and the model inference process. This process was also pre-established before model inference procedure.
Reproducibility	Data and scripts for reproducing all the results are available at https://github.com/hangzhangust/HCV NS3.
Randomization	This is not relevant to this study, as all data has been used to infer the model and conduct the analysis.
Blinding	This is not relevant to this study, as all data has been used to infer the model and conduct the analysis.

Did the study involve field work? ☐ Yes ☒ No

Reporting for specific materials, systems and methods

We require information from authors about some types of materials, experimental systems and methods used in many studies. Here, indicate whether each material, system or method listed is relevant to your study. If you are not sure if a list item applies to your research, read the appropriate section before selecting a response.

Materials & experimental systems

n/a	Involved in the study
☒	☐ Antibodies
☒	☐ Eukaryotic cell lines
☒	☐ Palaeontology and archaeology
☒	☐ Animals and other organisms
☒	☐ Clinical data
☒	☐ Dual use research of concern
☒	☐ Plants

Methods

n/a	Involved in the study
☒	☐ ChIP-seq
☒	☐ Flow cytometry
☒	☐ MRI-based neuroimaging
