# Supplementary material for: Direct-acting antiviral resistance of Hepatitis C virus is promoted by epistasis
Source: Nat Commun. 2023 Nov 17;14:7457. doi: 10.1038/s41467-023-42550-6 (PMC10656532; doi:10.1038/s41467-023-42550-6)
Supplement: Supplementary file 4 — Description of Additional Supplementary Files [file 41467_2023_42550_MOESM4_ESM.pdf]

<u>Description of additional supplementary files</u>

Supplementary Data 1: Infectivity measurements for NS3 that were used for correlating with predictions from the fitness landscape model.

Supplementary Data 2: Accession numbers of NS3 sequences used for inferring the model.

Supplementary Data 3: The mean escape time predicted by the in-host evolutionary model for each residue with DRMs
